# Harmonization and standardization of personalized oncology care within the German network for personalized medicine (DNPM): methods of a controlled observational study employing an adapted stepped-wedge design

**DOI:** 10.3389/frhs.2025.1684984

**Published:** 2026-01-02

**Authors:** Antonia Bauer, Sebastian Himmler, Nisar Malek, Lisa-Marie Brenner, Yvonne Möller, Stephanie Vigier, Markus Buchner, Leonie Sundmacher, Stefanie Joos, Peter Martus

**Affiliations:** 1 Institute of General Medicine and Interprofessional Care, University Hospital Tuebingen, Tuebingen, Germany; 2 Chair of Health Economics, Technical University of Munich, Munich, Germany; 3 Center for Personalized Medicine, University Hospital Tuebingen, Tuebingen, Germany; 4 Department of Internal Medicine I, University Hospital Tuebingen, Tuebingen, Germany; 5 Institute for Clinical Epidemiology and Applied Biometry, University Hospital Tuebingen, Tuebingen, Germany

**Keywords:** personalized medicine, oncology, network-based, interprofessional, mixed-methods analysis, stepped-wedge design

## Abstract

**Background:**

The goal of personalized medicine (PM) is to provide tailored diagnostics and therapies for individual patients, primarily in oncology. However, significant regional disparities exist in its implementation. The Deutsches Netzwerk für Personalisierte Medizin (DNPM; German Network for Personalized Medicine) project aims to harmonize and network the implementation of PM in Germany.

**Methods:**

The DNPM project is evaluated as a Hybrid Type 3 implementation study using a non-randomized, modified stepped-wedge design. Twenty-one university hospital sites across Germany will transition from standard, non-harmonized care to a harmonized personalized medicine intervention at different time points, based on administrative readiness indicated by certification. The primary outcome is change in patient management due to molecular tumor board (MTB) decisions, assessed on three tiers using prospectively collected primary data entered by clinical staff: (1) number of patients with access to an MTB, (2) proportion with revised diagnostic or therapeutic recommendations, and (3) proportion with implemented changes. Therefore, the study aims to include 4,807 patients (intervention group: 3.507). A mixed-methods approach is employed to evaluate further aspects of the implementation process including patient and staff satisfaction, perceived quality of care, and the degree of harmonization and collaboration within the network. Health economic outcomes include health-related quality of life, healthcare utilization costs, and intervention-related costs.

**Discussion:**

The study's wide range of outcome parameters and mixed-methods approach yield robust results for implementation insights. A limitation of the study design is the lack of a clear cutoff for transitioning from the control to the intervention group and the lack of randomization. Additionally, the health economic evaluation is limited by the absence of health insurance claims data, as no insurance company is involved in the project.

**Trial registration:**

This trial is registered with the German Clinical Trials Register (DRKS) under the trial number DRKS00031622 (registration date: 23 May 2023)

## Introduction

### Background and rationale

Personalized medicine (PM) leverages rapid advancements in high-throughput diagnostics, functional imaging, and data processing, including artificial intelligence, to provide patients with targeted treatments that minimize side effects. In oncology, the principles of molecularly stratified medicine are being extensively researched across all types of tumors and are already being applied in patient care (1–3). This has led to the segmentation of tumor entities into increasingly smaller patient groups that, despite having the same entity, differ significantly in their molecular structure and therefore require different treatments. Consequently, this has resulted in highly individualized treatment approaches, which must rely on an evidence-based, harmonized decision-making process that can only be achieved through the collection of individual disease and therapy data.

To support these advancements, four university hospitals in Baden-Württemberg (BW) (Tübingen, Freiburg, Ulm, and Heidelberg) have each established a Center for Personalized Medicine (ZPM), included as a center in the state hospital plan by the Baden-Württemberg Ministry of Social Affairs in 2019, aiming to integrate PM principles (such as the systematic integration of biological, genetic, environmental, and lifestyle variability into prevention and treatment strategies, with the aim of improving individual patient outcomes and reducing adverse effects) into the healthcare system. Although the centers initially focus on oncology, they are designed to adapt to other disease areas. A key element is the systematic collection of data from treatment contexts and linking it with data from sources such as gene sequencing, omics technologies, or imaging, to improve individualized treatment. This approach depends on harmonized diagnostics and treatment decision-making.

The implementation of the ZPM concept in BW, with a focus on molecular oncology, is based on significant preliminary work from precision oncology networks, including the Molecularly Aided Stratification for Tumor Eradication Research (MASTER) program of the German Cancer Research Center (DKFZ). This initiative developed key elements and standards, such as cross-entity and cross-location molecular tumor boards (MTBs), standardized documentation of longitudinal clinical data, grading of genetic variant evidence, and multisite molecularly stratified clinical trials, among other translational research projects (4, 5). Furthermore, the National Genomic Medicine Network (nNGM) for lung cancer, established in 2016 across all 13 comprehensive cancer centers (CCCs), has contributed to the harmonization of diagnostics, documentation, and MTB recommendations for lung cancer (6).

Building on these efforts and the existing structures within CCCs, the ZPM-BW initiative, in collaboration with health insurance providers in BW [Allgemeine Ortskrankenkasse (AOK) and Verband der Ersatzkassen (vdek)], has developed a specialized concept that establishes shared quality standards and integrates ZPM principles into routine care. Quality criteria have been defined across the following areas:

- Structural and organizational setup of the ZPM and its network
- Decision-making processes in MTBs
- IT systems
- Documentation
- Quality management

Based on data harmonization processes introduced within ZPM-BW, all CCCs in Germany will now be enabled to integrate their structures and processes into the emerging DNPM. In this project, the new processes and structures outlined above will be implemented at CCCs across Germany, establishing further ZPMs. By the end of the project's funded period, a nationwide, digitally interconnected, interoperable, and patient-centered network will be in place to translate PM into patient care (7). This will considerably increase the amount of data, utilize the experience of 13 top centers, and significantly improve the possibility of comprehensive care in personalized oncology. To exchange this data (e.g., demographic patient data, diagnoses, treatment courses, genetic and pathological phenotype, recommendations from the MTB, and follow-up on these recommendations, as well as outcome data), the DNPM is creating a nationwide data platform, the dnpm:DIP, to enable secure, pseudonymized sharing of phenotypic, genomic, and imaging data between centers. This decentralized system supports quality control by national health insurers and facilitates data-driven decision-making in MTBs and patient inclusion in molecularly stratified trials (7).

To guarantee a comparable quality of innovative and interdisciplinary care across all centers, the DNPM has set up a certification system for the ZPM in the DNPM with the German Cancer Society [Deutsche Krebsgesellschaft (DKG), OnkoZert] and thus serves as a blueprint for clinical implementation.

### Specific project goals at the micro-, meso-, and macro-levels

Micro-level: On the micro-level (individual), the DNPM will introduce and further develop standardized quality criteria and digital documentation procedures, including the following:

- Inclusion criteria for MTBs
- Molecular diagnostics and bioinformatic analysis methods (shared genetic core data set, ring trials for the analysis pipeline)
- Evidence determination for therapy recommendations
- Digital documentation (interoperable, standardized data models)
- Imaging as follow-up monitoring (digitally, structurally, and machine-readable documented)

Meso-level: Establishing ZPMs at all sites involves setting up fundamental structures (ZPM office and board) and integrating these into the respective university hospital and CCC. A major milestone is the development of a regional care and data network that ensures cost-efficient implementation and patient flow management and prevents uncontrolled diffusion of PM, multiple treatments, and over-provision of care.

Macro-level: Networking the ZPMs into DNPM governance structures and sustainability plans will be developed in consultation with all partners, and a DNPM steering board will be established with equal representation from each ZPM. A network-wide database (dnpm:DIP) will be created in collaboration with existing national infrastructure initiatives, which will be essential for the utilization of patient data, especially as patients are increasingly stratified into smaller cohorts.

Through these structured documentation and data exchange efforts, a robust evidence base will be built, serving as the foundation for more efficient clinical trials and improved patient outcomes in PM.

### Objectives

#### Specific objectives for primary analysis

- Increase the number of patients for whom standardized access to the new form of care of personalized diagnostics and therapy is possible
- Increase the chance of treatment recommendations
- Increase actual implementation of treatment recommendations

### Specific objectives for secondary analysis

- Patients can benefit from the standardization of PM in ZPM regarding access to individualized therapy.
- The ZPM specialist concept BW can be transferred to other locations.
- Increased communication between the individual ZPMs provides added value for the efficiency of patient care.

## Methods: participants, interventions, and outcomes

### Intervention description

All participating centers will undergo a certification process as oncological centers in accordance with the requirements set by the German Cancer Society (8). Centers will transition to the intervention group once they meet the DNPM criteria and receive a positive audit result. In the intervention group, patients will be treated according to DNPM standards, which encompass quality-assured molecular diagnostics and evidence-based treatment recommendations within interdisciplinary MTBs. The therapies initiated at these sites may vary individually in terms of timing, duration, and type.

The DNPM standards encompass ongoing quality control measures, the standardization of diagnostics, bioinformatics, MTBs, referrals for genetic counseling and imaging, and clinical decision-making based on harmonized evidence levels. See Appendix 1 for a summary of the dimensions of certification. The standardized and registered components of ZPM include a uniform and organized documentation of MTB decisions (approximately 40 harmonized parameters), coordinated clinical and genomic datasets (each containing over 200 parameters), consistent follow-up and response documentation, and a structured process for off-label treatment applications that has been agreed upon with the medical service and government health insurances. A nationwide network in the field of personalized oncology is established following these core measures:

- **Micro-level:** improvement of evidence-based treatment recommendations in the MTB
- **Meso-level:** quality-assured, harmonized operation of ZPM (Centers for Personalized Medicine) at CCC locations
- **Macro-level**: establishment and use of a digitally connected PM registry (using the example of the Baden-Württemberg ZPM network) to improve treatment recommendations

### Trial design

The current study employs a Hybrid Type 3 design, evaluating the overall implementation while monitoring and collecting data on the clinical intervention's effects on important outcomes (9). Using a mixed-methods approach, the study integrates quantitative measurements and qualitative data collection across multiple stakeholder groups, including patients and healthcare staff. In addition, key structural and process parameters will be documented over time, and a health economic evaluation will be conducted. The study design is based on a modified stepped-wedge approach: The participating centers transition at different time points from a control condition—characterized by site-specific, non-harmonized PM practices—to the intervention condition, which reflects harmonized PM procedures. While this approach resembles the classical stepped-wedge design in its sequential rollout across sites, it deviates in one important aspect: The timing of the transition is not randomized. Instead, it is determined by administrative and organizational readiness. Specifically, a center enters the intervention group on the date documented in its certification form, which confirms that essential steps toward PM harmonization have been implemented. The certification criteria align with the standards set by the German Cancer Society (Deutsche Krebsgesellschaft) (8).

### Timeline of the study

Participant recruitment was completed on 30 September 2024. Data collection is expected to be finalized by the end of November 2025, with results anticipated in January 2026.

### Study setting

Patients are recruited at study sites scattered throughout Germany and partly organized in local alliances. These study sites usually represent existing comprehensive cancer centers attached to university clinics (*N* = 21). The DNPM originated from a regional initiative in Baden-Württemberg that began in 2015, which initially aimed at establishing Centers for Personalized Medicine (ZPM) at the University Medical Centers located in Freiburg, Heidelberg, Ulm, and Tübingen.

A list of study sites throughout Germany is presented in Table 1.

**Table 1:** A list of study sites throughout Germany.

Alliance	Study site
CCC CIO-ABCD	Aachen (DNPM)
CCC Berlin	Berlin (DNPM)
CCC CIO-ABCD	Bonn (DNPM)
NCT/UCT Dresden	Dresden (DNPM)
CCC CIO-ABCD	Düsseldorf (DNPM)
CCC Essen-Muenster	Essen (DNPM)
CCC Frankfurt—Marburg	Frankfurt (DNPM)
ZPM Freiburg	Freiburg (DNPM)
CCC Niedersachsen	Goettingen (DNPM)
CCC Hamburg	Hamburg (DNPM)
CCC Niedersachsen	Hannover (DNPM)
ZPM Heidelberg	Heidelberg (DNPM)
CCC CIO-ABCD	Koeln (DNPM)
CCC Mainz	Mainz (DNPM)
CCC Frankfurt—Marburg	Marburg (DNPM)
CCC Muenchen	Muenchen LMU (DNPM)
CCC Muenchen	Muenchen TU (DNPM)
CCC Essen-Muenster	Muenster (DNPM)
ZPM Tuebingen	Tuebingen (DNPM)
ZPM Ulm	Ulm (DNPM)
CCC Wuerzburg	Wuerzburg (DNPM)

### Sample size

The study aims to recruit a total of 4,807 patients, including 3,507 in the intervention group and 1,300 in the control group. The following assumptions underlie the sample size estimation: For nine newly established ZPMs (centers), two centers would be acceptable as dropouts. At least four existing centers in BW are considered unlikely to drop out, and seven newly initiated ZPMs serve as the basis for the sample size estimation. The new centers will contribute to controlling/intervening patients before/after initiation as ZPM, while the existing ZPM will only have intervention patients. This approach, inspired by the stepped-wedge design, has already been successfully used in several projects funded by the Innovation Fund (10). An average stay of 6 months in the control phase is assumed. It is estimated that each comprehensive cancer center (CCC) will have between 300 and 500 patients with PM, resulting conservatively in 1,300 control patients. This is compared with an estimated 3,500 ZPM patients from existing ZPM and those newly implemented during the study period. With these numbers, ignoring the cluster effect, differences of approximately 5% can be detected in the chi-squared test (Type I error 5% two-sided) with a power of >80% (exactly 86%) and differences of 7% with >90% (exactly 96%) power. Assuming a variance inflation due to cluster effects of 2, differences of 7% can still be detected with 85% power, and with very high cluster effects of 4 or 6, differences of 10% or 13% can be detected with 85% power.

### Recruitment

Patients are recruited by staff at the cancer centers according to predefined inclusion and exclusion criteria. The consortium lead is implementing strategies for adequate participant enrolment by actively supporting centers in the establishment of adequate structures and by regularly providing feedback on recruitment data provided by the evaluation team. Recruitment for interviews will continue until thematic saturation is reached, guided by the concepts of thematic saturation (11) and information power (12).

#### Eligibility criteria

Patients with severe, advanced cancer who have undergone guideline-conformant therapy and those with rare tumors for which no guideline-conformant therapy exists.

Inclusion criteria:

- Diagnosis of cancer, including progression or recurrence (all cancer types can be included)
- Patients are capable of participating in data collection
- Treatment at one of the participating CCC/ZPMs or patients seeking a second opinion there
- Molecular diagnostics are possible
- Patient qualifies for the MTB of the center
- First presentation at the MTB of the center
- Patients aged 18 years or older
- Informed consent—written consent for participation in the study and data sharing

#### Exclusion criteria

- Language or cognitive impairments that prevent data collection even with support

### Interventions

#### Explanation for the choice of comparators

The comparator consists of centers that have not yet completed the certification process to reflect the state of care prior to certification and to show the improvement in process-related and medical outcomes once the DNPM standard is established in the respective cancer center. Control patients at the sites meet the criteria for inclusion in the MTB but are not certified according to the standards of the DNPM in the MTB. The DNPM standard includes quality-assured molecular diagnostics as well as evidence-based treatment recommendations in interdisciplinary MTBs. Therapies initiated at the sites may vary individually by timing, duration, and type.

### Criteria for discontinuing or modifying allocated interventions

The trial will be discontinued for a given trial participant in case the patient withdraws his or her informed consent or in the case of his or her death. Modification of interventions is not applicable as the intervention is implemented in centers, not in specific patients.

### Strategies to improve adherence to interventions

Regular meetings will be held with study staff from all participating centers to improve data collection and documentation. Feedback from the centers will also be used to improve the design of the database and its interface. Besides this, all steps of the data collection will be regularly outlined at steering committee meetings, where all centers are present to ensure that centers understand the purpose of questionnaires, interviews, focus groups, etc. Next to this, updates on case numbers will be given regularly in these meetings to ensure that centers meet their recruitment goals. Additionally, questionnaires will be piloted with study staff in order to ensure that these are relevant to their daily work and are well understood.

### Outcomes

A central element is the primary endpoint at the patient level (micro-level), which is divided into a three-tiered endpoint and allows conclusions about the benefits of the intervention. Tier 1 includes the frequency of access to the new form of care; Tiers 2 and 3 encompass the development and implementation of therapy proposals. The quantitative evaluation is supplemented by a quantitative patient survey using validated questionnaire instruments and a health economic assessment. In addition to quantitative data collection, interviews with patients are conducted to capture patient understanding of and expectations regarding personalized medicine. Overall, the development and enhancement of structures and processes in centers for personalized medicine (ZPM) is documented through standardized self-report questionnaires (see Appendix 2), as well as interviews and focus groups. This also specifically captures the exchange and knowledge transfer between ZPMs, as well as the extent to which an exchange of harmonized data has taken place. A complete list of the measures used in the quantitative and qualitative process evaluation, as well as secondary patient-related endpoints (clinical: response, PFS, OS, toxicity, patient questionnaires, and health economic measures), is presented in Table 2. A design diagram further elaborating on the data collection time points can be found in Appendix 3.

**Table 2:** Primary and secondary endpoints and data collection.

Level	Evaluation	Primary endpoint	Time point	Instrument
Micro-level	Quantitative evaluation	Change in patient management based on the decision of the tumor board (assessed on three tiers):		Based on a quantitative questionnaire filled out by staff on site *(included in the provider questionnaire)*
		Tier 1: number of patients admitted to the tumor board	T0 = day of initial presentation at the tumor board)	
		Tier 2: proportion of patients with changed diagnostic strategy and/or therapy recommendation due to the decisions of the tumor board	T1 = day of therapy decision	
		Tier 3: proportion of patients with an actually implemented changed diagnostic strategy and/or therapy	T2 = day of the first therapeutic intervention	
		Secondary endpoints		
Micro-level	Quantitative evaluation	Clinical data of patients treated in the MTB • Response • Progression-free survival • Overall survival • Toxicities	event-driven	*Included in the provider questionnaire*
		Patient satisfaction and quality of life of patients treated in the MTB • Satisfaction • Quality of life	At study enrollment, 2–3 months after enrollment, and 5–6 months after enrollment	• Modified questionnaire instrument based on the PSCC-G instrument • Established questionnaire instruments for disease-specific quality of life, EQ-5D-5L, and EORTC QLQ-C30 *(included in the patient questionnaire)*
		Health economics • QALYs • Total costs (healthcare costs, intervention costs) • ICER	5–6 months after enrollment	• EQ-5D-5L • Modified FIMA questionnaire • Controlling the data of participating university hospitals • Survey of centers *(included in patient questionnaire)*
	Qualitative evaluation	Patient-level survey: • Understanding and attitude toward personalized medicine • Qualitative assessment of the implementation processes • Expectations regarding care provided by the personalized medicine services (ZPMs)/the decentralized personalized medicine (DNPM) • Perceived care before and during treatment with ZPMs/DNPM • Costs incurred in the context of personalized medicine care	At a later stage in the project	Semi-structured interviews with patients
Meso-level and macro-level	Process evaluation (quantitative)	Perspective of staff from centers for personalized medicine (ZPMs): • Degree of standardization of processes • Establishment of new coordination processes at the medical or professional level (e.g., expert committees) • Implementation and structural costs (costs incurred from transitioning to ZPM/DNPM) • Process costs (ongoing costs before ZPM, or of the individual ZPMs) • Exchange/knowledge transfer between ZPM • Exchange of harmonized data • Presence of interconnected data structures (e.g., completeness, quality) • Exchange of harmonized data between the ZPMs • Communication and decision-making structures and processes	• T0: at the time of certification • T1: at an intermediate stage in the project • T2: at a later stage in the project	Quantitative questionnaire based on the certification forms at the center, participation in meetings of the MTB working group, and analysis of the working group’s meeting minutes *(organizational structure and process survey)*
	Process evaluation (qualitative)	Perspective of staff from centers for personalized medicine (ZPMs): • Attitudes and experiences regarding and with personalized medicine • Attitudes toward and experiences with the change processes • Satisfaction of the involved staff • Planning and adaptations in the implementation process • Possible barriers and facilitators in the implementation process • Perceived added value of the new form of care • Exchange/knowledge transfer between ZPM • Exchange of harmonized data • Presence of interconnected data structures (e.g., completeness, quality) • Exchange of harmonized data between the ZPMs • Communication and decision-making structures and processes	At an intermediate stage in the project	Semi-structured interviews with staff from centers for personalized medicine, participation in meetings of the MTB working group, and analysis of the working group’s meeting minutes
	Process evaluation (qualitative)	Perspective of staff from centers for personalized medicine (ZPMs): • Planning and adaptations in the implementation process • Possible barriers and facilitators in the implementation process • Perceived added value of the new form of care	At a later stage in the project	Provider focus groups

### Participant timeline

Table 3 shows the participant schedule throughout the intervention period.

**Table 3:** Participant schedule.

Specification of data collected	Participant schedule			
	Enrolment	2–3 months post-enrolment	5–6 months post-enrolment	Close-out
Time point/assessment	T0	T1	T2	T3
Study participation				
Eligibility screen	X			
Informed consent	X			
Allocation	X			
Clinical data collection				
Socio demographics	X			
Diagnose	X	X	X	
(Further) therapies in the last 3 months outside the MTB recommendation	X	X	X	
MTB recommendations		X	X	
Follow-up on MTB recommendations		X	X	
Scheduled end (follow-up completed after 5–6 months)				X
Reason for premature end				X
Patient questionnaire				
Socio demographics	X	X	X	
Living situation	X	X	X	
Financial Situation	X	X	X	
Social support	X	X	X	
EQ-5D-5L (health-related quality of life)	X	X	X	
EORTC QLQ-C30 (disease-related quality of life)	X	X	X	
FIMA (Utilization of health services)	X	X	X	
Patient satisfaction	X	X	X	
Patient interviews				
Understanding, expectations, and attitudes toward PM, perceived care		X	X	

### Data collection and management

#### Quantitative evaluation

The primary endpoint contains three tiers. At Tier 1, the primary endpoint is the number of patients who receive an indication-driven, standardized access to the new form of care—personalized diagnostics and therapy—defined as access to the MTB. For this endpoint, a standardized data collection instrument will be developed to document both the original procedure and the newly implemented one, including the extent of the changes made (e.g., additional molecular diagnostics and therapy). The new procedure will be based on predefined, evidence-based criteria established by the ZPM network in BW. At Tier 2, it will be assessed for which patients the diagnostic process or diagnostic interpretation was significantly changed. Among this group, Tier 3 evaluates the proportion of patients for whom the changes ultimately led to treatment within the framework of personalized medicine. These changes may also include recommendations for genetic counseling, which—although not directly involving therapy or additional diagnostics—represent a modification in patient care.

Secondary clinical endpoints will be assessed continuously during the study: Adverse events (AEs) and serious adverse events (SAEs) will be recorded as they occur, and overall survival (OS) and progression-free survival (PFS) will be recorded every 6 months (T4–T8) until the end of the observation period, with a minimum follow-up of 6 months and an average follow-up of 12 months.

#### Process evaluation

##### Qualitative share of the process evaluation

Semi-structured patient interviews: At the patient level, individual interviews with patients will complement the quantitative data collection. These interviews will focus not only on the qualitative assessment of processes from the patient's perspective but also on understanding their attitudes and perceptions toward patient management (PM). A total of 10–15 interviews with patients from various locations will be conducted, using a stratified selection based on age, gender, center, and condition throughout the entire intervention phase.

Semi-structured provider interviews: To identify possible obstacles and facilitating factors in the implementation process, qualitative surveys will be conducted in the form of individual interviews (*N* = 21) and focus groups (*N* = 3–4) with persons holding central roles (ranging from administrative, coordinative roles to medical roles) in the cancer centers toward the end of the implementation phase. The aim is to include staff from all participating centers in the interviews.

##### Quantitative share of the process evaluation

Organizational structure and process survey: The qualitative analysis will be supplemented by a self-report questionnaire in accordance with the certification requirements of the German Cancer Society (Deutsche Krebsgesellschaft) (8). To assess the level of establishment of the individual ZPMs, the achievement of goals during the implementation of functional areas and their integration into the ZPM will be measured. This includes an established telemedicine access for external practitioners, quality-assured molecular diagnostics, and evidence-based therapy recommendations in interdisciplinary molecular tumor boards (MTBs).

In addition, a range of patient-reported outcomes (PROs) will be assessed. These include generic quality of life, measured using the standardized EQ-5D-5L questionnaire, and disease-specific quality of life, assessed with the EORTC QLQ-C30. An additional secondary PRO is treatment-related patient satisfaction, evaluated using the Patient Satisfaction with Cancer-related Care questionnaire (PSCC-G), which has been validated in German (13). All PROs will be collected at three individual time points: at the start of therapy (T2, baseline for secondary endpoints), after 3 months (T3), and after 6 months (T4).

#### Health economic evaluation

For the purpose of the health economic evaluation, health-related quality of life, mortality, and cost data have to be collected for intervention and control patients. Quality of life is assessed based on patients' responses to the validated EQ-5D-5L questionnaire at study enrollment, 2–3 months after enrollment, and 5–6 months after enrolment (14, 15). Deaths are recorded in the questionnaire filled out by site staff. Inpatient treatment costs incurred at the university hospitals are extracted from the respective controlling departments. If the cancer treatment of patients does not take place at the study center itself, costs are approximated by the type of therapy recorded in the study documentation and the respective averages based on the then available controlling data from other patients. To capture other healthcare costs incurred outside the included university hospitals, an adjusted version of the FIMA questionnaire (questionnaire on the use of medical and non-medical health and care services in later life), a validated survey instrument for healthcare utilization for the German context (16, 17), is administered to patients at study enrollment, 2–3 months after enrollment, and 5–6 months after enrollment. Costs of the DNPM intervention itself are approximated through assessments made by coordinators of the study centers (a) in a quantitative survey (as part of the process evaluation questionnaire) and (b) in a focus group with the cost items of the DNPM project's overall budget as a starting point.

## Analysis and anticipated results

### Quantitative evaluation

The hierarchically organized endpoints described above (see Table 2) will be analyzed as binary variables (yes/no) while accounting for cluster effects from the individual centers using generalized estimating equations (GEE) in a logistic regression model (working correlation structure: exchangeable). The study arm (traditional procedure vs. standardized procedure according to the new form of care) will be coded as a binary variable, changing over time for centers newly included in the intervention condition. The Type I error rate will be set at 0.05 (two-sided). *P*-values for the secondary endpoints will be reported, but these should not be interpreted confirmatively. No corrections for multiple testing are necessary. Cluster effects will be addressed using GEE. In the primary analysis, we will use the independence estimation equation, and in a secondary analysis, we will use the exchangeable correlation structure within centers which allows for estimation of the cluster effect.

We expect the intervention to have a measurable impact on patient management as reflected in the primary outcome. Specifically, we anticipate the following effects across three levels of outcome assessment: Level 1, an increase in the frequency of indication-driven, standardized access to the MTB following implementation of the intervention; Level 2, a higher proportion of cases in which the recommendation for further diagnosis or therapy is modified as a result of MTB discussions under the harmonized approach; Level 3, an increase in the proportion of patients for whom the recommended changes in diagnostics or therapy are actually implemented in clinical practice. These analyses (Levels 1–3) will be additionally done with respect to the evidence level of the recommended therapies.

These expected changes reflect the underlying hypothesis that harmonization and standardization of personalized oncology care will enhance the integration of MTBs into clinical workflows and improve outcomes for patients. However, we have to take into account that harmonization is a complex process and implementation is not done on a certain date. Thus, in secondary analyses, we will exclude patients in the last 3 and 5 months before certification from the control group, and in an additional secondary analysis, we will analyze the time trend in centers that were newly certified, correcting for the time trend in centers that had been certified already at the beginning of the project. AEs and SAEs are expected to remain within acceptable limits, indicating good tolerability. Additionally, we expect improvements in OS and PFS, among patients receiving targeted therapies based on molecular tumor profiling. These outcomes would support the clinical value of the intervention in routine care.

### Process evaluation

#### Quantitative component of the process evaluation

Quantitative questionnaires will be analyzed using descriptive analyses following scaling and observed data distribution. Thus, means and standard deviations will be given for normally distributed data and medians and interquartile ranges for non-normally distributed data. Categorical data will be reported by absolute and percentage frequencies. Descriptively, different professions and the different study centers will be compared.

#### Qualitative component of the process evaluation

All qualitative data will be transcribed, pseudonymized, and analyzed using qualitative content analysis according to Kuckartz (18), adhering to established methodological standards and quality criteria. Initially, a coding frame will be developed based on the sighting of 2–3 transcripts. From this, a codebook will be developed that will be applied to code the remaining transcript. All transcripts will be independently analyzed by two experienced qualitative researchers. Any disagreement in the resulting coding will be discussed, and the extent to which the independent coders agreed or disagreed will be reported descriptively (19).

The resulting category system will provide additional information to the questionnaire data and help to better understand the perspectives of patients and care providers in the field of personalized medicine.

In addition to the primary outcome, we anticipate several important findings related to the secondary endpoints across micro-, meso-, and macro-levels of the healthcare system. At the micro-level, we expect that patients will perceive benefits from the standardization of PM within the ZPMs, particularly through more consistent and equitable access to individualized therapy options. These benefits will be evaluated using a range of patient-centered outcomes, as outlined in Table 2. At the meso-level, we aim to assess the transferability of the ZPM specialist concept developed in BW to other participating sites. We anticipate identifying key facilitators and barriers to successful implementation, as well as necessary modifications to support context-specific adaptation.

Finally, at the macro-level, we expect that improved communication and collaboration among ZPMs within the national network will generate added value by enhancing patient care delivery. This may include greater harmonization of clinical structures and workflows, improved interoperability of data systems, and coordinated decision-making and resource sharing. These anticipated findings will inform future strategies for scaling and sustaining harmonized PM practices across healthcare institutions.

### Health economic evaluation

In the health economic analysis, treatment costs and intervention costs will be compared with quality-adjusted life years (QALYs) in a cost–utility analysis for both the intervention and control groups and summarized as an incremental cost-effectiveness ratio (ICER). The ICER reflects the relationship between the additional costs incurred through the ZPM (intervention) and the demonstrated benefit in terms of QALYs.

Quality of life will be assessed using the standardized EQ-5D-5L questionnaire at the start of therapy (T2; baseline), at 6 weeks (T3), and at 6 months (T4) after therapy initiation. These data will be translated into quality-adjusted life years. As cost parameters, the analysis will consider all implementation costs related to establishing the ZPMs at the intervention sites, as well as all treatment-related costs associated with PM in both the intervention and control groups. For this purpose, data will be collected from the controlling units of participating institutions that deliver PM, which provide patient-level information on cost coverage by statutory health insurance (SHI).

Since only a portion of patients are treated directly at the respective centers (in some ZPMs, <50%), and because outpatient costs are not centrally documented within hospital controlling systems—making comprehensive cost capture difficult—additional patient-level data will be collected through the following methods:

Patient self-reports on healthcare utilization will be collected based on an adapted version of the FIMA questionnaire, administered at 3 and 6 months after therapy initiation. Costs of implemented MTB recommendations (primary endpoint, Level 3) will be calculated based on the reported pharmaceuticals and therapy modalities (e.g., using data from the Lauer-Taxe drug price database). To account for process and structural costs associated with care delivery in the ZPMs, focus groups with participating centers will be conducted at the beginning of the project. These will identify both the additional costs incurred through the transition to ZPM-based care and the potential cost savings expected as a result of the structural change.

The analysis will be conducted from the perspective of SHI. The findings will be evaluated for their transferability to the broader SHI-insured population. Statistical uncertainty around the ICER will be illustrated and estimated using cost-effectiveness acceptability curves (CEACs).

At the macro-level, we anticipate that increased communication and coordination between the individual ZPMs will lead to measurable improvements in the efficiency of patient care across the network. These improvements are expected to manifest as cost savings through greater harmonization of clinical structures and processes and enhanced interoperability of data systems. Through the health economic evaluation, we aim to quantify these effects by comparing implementation and treatment-related costs between intervention and control settings, while also capturing structural and process-related cost changes associated with the network-wide adoption of personalized medicine. We expect that the findings will demonstrate that a well-connected ZPM network not only improves care delivery but also supports more efficient resource utilization, with potential implications for cost-effectiveness at the system level.

### Data integration

Within the process evaluation, data integration will follow a convergent mixed-methods design. Qualitative and quantitative data will be analyzed both interactively and in parallel, depending on the specific evaluation component, and merged during the interpretation stage (20). Integration will focus on identifying consistencies, complementarities, and discrepancies between data sources. From the provider’s perspective, different methodological approaches will be triangulated. An interactive analysis will be conducted in which the provider questionnaire informs the design and focus of subsequent provider interviews, and the insights gained from these interviews will, in turn, guide the development of the focus groups.

### Methods for additional analyses (e.g., subgroup analyses)

#### Quantitative evaluation

Secondary analyses will be done adjusted for the characteristic “rare tumors yes/no,” as described in the inclusion criteria, as well as for the factor “urban/rural,” which arises from the regional structure of the participating CCCs. Planned subgroup analyses will be conducted for the individual CCCs, the type of tumors (rare: yes/no), and the demographic characteristic of urban vs. rural.

#### Process evaluation

No sensitivity analysis is planned for the process evaluation. There is currently no subgroup analysis planned; this decision will be reevaluated during the evaluation process.

#### Health economic evaluation

Sensitivity analyses, for example, relating to different definitions of the intervention costs, are planned for the health economic evaluation. The same subgroups as in the quantitative evaluation are also considered here.

### Methods in analysis to handle protocol non-adherence and any statistical methods to handle missing data

#### Quantitative evaluation

We differentiate between lack of adherence to the intervention and lack of adherence to the evaluation. Adherence to the intervention is part of the evaluation and will be analyzed but not adjusted in the analyses of outcomes. Non-adherence regarding the evaluation will be addressed by incorporating covariates that reflect relevant aspects of non-adherence into the regression models section. Additionally, separate analyses will be conducted for observational groups with varying levels of adherence. Any differences in outcomes between these groups, as well as the potential role of adherence as a predictor of treatment effectiveness, will be transparently analyzed and reported.

Assuming that missing data can be reasonably treated as missing at random, multiple imputation techniques will be employed to minimize the loss of valuable non-missing information that could occur with other methods, such as inadequate listwise deletion. We will use 500 imputation samples, study center, and center status (control vs. intervention) as predictors. Furthermore, we will consider diagnostic classes and measures of disease severity as potential predictors in the imputation model.

##### Process evaluation

The non-compliance with the protocol will not be handled, as there is no underlying protocol that needs to be followed as part of the data collection process. Instead, a potential non-compliance in the context of the certification process (necessary to achieve the DNPM standard) represents an important outcome within the process evaluation.

##### Health economic evaluation

Protocol non-adherence and missing data in the health economic evaluation will be handled in line with the approaches described for the quantitative evaluation.

## Discussion

The objective of this project is to establish a nationwide, digitally interconnected, interoperable, and patient-centered network for personalized oncology care. This is particularly beneficial for cancer patients, who often require highly individualized treatment strategies.

The study employs a modified stepped-wedge design, which enables a gradual, center-specific transition from standard, non-harmonized PM practices (control condition) to harmonized, quality-assured PM (intervention condition). In contrast to classical stepped-wedge trials, the timing of each center's transition is not randomized but is instead determined by administrative readiness, as marked by the date of certification. This approach accounts for inter-center heterogeneity and allows institutions to implement the intervention at their own pace, making the design both flexible and pragmatic in a real-world healthcare context.

However, the lack of randomization and a distinct, uniform cutoff point for transitioning from control to intervention introduces complexity in the evaluation. The gradual implementation process may lead to variation within the control condition, which could affect comparability across sites and time points. To address this, we will systematically document and analyze this heterogeneity as part of the evaluation strategy. Moreover, as described in the Methods, we will perform several sensitivity analyses to account for the fact that transition is an entire process and not implemented at a certain date in each center.

The study also benefits from a mixed-methods approach, combining quantitative outcome measures with qualitative data from key stakeholders, including patients and healthcare professionals. This methodological diversity supports the generation of robust, context-sensitive insights into the implementation process.

Additional challenges include the risk of selection bias in patient interviews, as participation may skew toward patients in better health. To mitigate this, the characteristics of interviewed patients will be compared with those of the broader study population. A further limitation is the absence of health insurance claims data for the health economic analysis, as no statutory health insurance providers are part of the project consortium. While alternative data sources on treatment and healthcare utilization will be used, these cannot capture the full spectrum of SHI-related costs at the same quality as claims data. We rely on self-reported data regarding the utilization of outpatient care, medical remedies and medical aids, long-term care, and rehabilitation services. While the FIMA showed promising results in the validation study regarding the accuracy of the reported utilization, self-reports, especially in this highly affected and vulnerable patient group, may suffer from higher attrition, recall bias, and lower accuracy. If sicker patients are less likely to fill in the FIMA questions, this introduces attrition bias in the corresponding cost estimates. Furthermore, we use information from the MTB recommendations and whether these were implemented to quantify costs for medications, instead of data from claimed prescriptions. While the former more accurately reflects exactly which specific treatments were implemented as a result of the MTBs, the latter would cover all SHI-covered medications prescribed to that patient. As such, our medication cost estimate will underestimate the full medication costs. At the same time, incremental cost differences regarding personalized medications can still be calculated. We obtain full inpatient cost data for all patients who are treated at the included university hospitals from the corresponding controlling departments—these are based on the same exports as inpatient claims data. However, as outlined above, there is a considerable share of study participants who are not treated at the university hospital where they were recruited, and the MTBs take place. This hampers the generalizability of inpatient costs to hospital cases outside of the included university hospitals, and as such, the validity of cost-effectiveness results for external patients.

Finally, the evaluation must address several implementation-related challenges, including the potential for confusion between elements of the intervention and aspects of the evaluation (e.g., mistaking data collection activities for part of the intervention itself), iterative modifications to the intervention during the study period (which can make it difficult to attribute outcomes to the intervention), and regulatory differences across participating centers (such as varying ethics approval processes and data protection requirements).

Despite these challenges, the study is expected to deliver valuable insights into the processes and outcomes of implementing harmonized personalized medicine in routine cancer care. The findings will inform future implementation efforts and may serve as a foundation for broader research initiatives in this field.

## Data Availability

The original contributions presented in the study are included in the article/Supplementary Material, further inquiries can be directed to the corresponding author.
